# Electrically Conducting Hydrogel Graphene Nanocomposite Biofibers for Biomedical Applications

**DOI:** 10.3389/fchem.2020.00088

**Published:** 2020-02-27

**Authors:** Sepehr Talebian, Mehdi Mehrali, Raad Raad, Farzad Safaei, Jiangtao Xi, Zhoufeng Liu, Javad Foroughi

**Affiliations:** ^1^Intelligent Polymer Research Institute, University of Wollongong, Wollongong, NSW, Australia; ^2^Illawarra Health and Medical Research Institute, University of Wollongong, Wollongong, NSW, Australia; ^3^Department of Mechanical Engineering, Technical University of Denmark, Lyngby, Denmark; ^4^School of Electrical, Computer and Telecommunications Engineering, Faculty of Engineering and Information Sciences, University of Wollongong, Wollongong, NSW, Australia; ^5^School of Textile Engineering, Zhongyuan University of Technology, Zhengzhou, China

**Keywords:** biopolymer, hydrogel, electrically conductive hydrogel, graphene, wet spinning, nanocomposite, biofibers

## Abstract

Conductive biomaterials have recently gained much attention, specifically owing to their application for electrical stimulation of electrically excitable cells. Herein, flexible, electrically conducting, robust fibers composed of both an alginate biopolymer and graphene components have been produced using a wet-spinning process. These nanocomposite fibers showed better mechanical, electrical, and electrochemical properties than did single fibers that were made solely from alginate. Furthermore, with the aim of evaluating the response of biological entities to these novel nanocomposite biofibers, *in vitro* studies were carried out using C2C12 myoblast cell lines. The obtained results from *in vitro* studies indicated that the developed electrically conducting biofibers are biocompatible to living cells. The developed hybrid conductive biofibers are likely to find applications as 3D scaffolding materials for tissue engineering applications.

## Introduction

Soft and flexible conductors are essential materials for bioelectronics that can be potentially implemented in a broad range of biomedical applications, ranging from cardiovascular (Shin et al., 2014, 2016; Navaei et al., 2016; Wang L. et al., 2017; Hu et al., 2019), muscle (Sasaki et al., 2014; Chen et al., 2015; Annabi et al., 2016; Jo et al., 2017; Guo et al., 2019), and nerve tissue engineering (Yang et al., 2016; Liu X. et al., 2017; Wang S. et al., 2017; Zhou et al., 2018) to implantable or wearable biosensors for human health monitoring (Mehrali et al., 2018; Kadumudi et al., 2019). In this context, conductive hydrogels are one of the most promising soft conductors owing to their high water content and molecular similarity to the natural soft tissues (Kim S. et al., 2015; Wu et al., 2016; Han et al., 2017; Qu et al., 2019). Conductive hydrogels are commonly composed of inherently conducting polymers such as polypyrrole (PPy) and polyaniline (PAni); however, these polymers often suffer from weak mechanical properties and poor biocompatibility and processability, which further hinder their biomedical application (Foroughi et al., 2009, 2011, 2015; Kaur et al., 2015; Mirabedini et al., 2016b). Thus, incorporation of conducting nanomaterials (such as graphene, carbon nanotubes, and gold nanoparticles) into hydrogels has emerged as an alternative approach to yield mechanically robust conductive hydrogels with enhanced biocompatibility (Mirabedini et al., 2016a; Mehrali et al., 2017; Wu et al., 2017; Liang et al., 2019). Nevertheless, the electrical conductivity of nanocomposite hydrogels is hampered by factors such as random distribution of nanomaterials in the polymeric network, and consequently efforts have been made to address this issue by assembling the nanomaterials into a macroscopically ordered structures within the hydrogel network (Ahadian et al., 2014, 2017; Shin et al., 2015; Luo et al., 2018). In this direction, wet spinning has emerged as a simple yet high-throughput assembly technique to transform carbon-based nanomaterials into fibers with highly ordered structures (Foroughi et al., 2012, 2016; Xu and Gao, 2014; Apollo et al., 2015; Mirabedini et al., 2015; Lu et al., 2017). The distinguishing factor of these wet-spun fibers is their flexibility in design, as they can be applied to common textile manufacturing processes (braiding, weaving, and knitting) to fabricate 3D structures and scaffolds (Akbari et al., 2014; Wang et al., 2015).

Among various carbon-based nanomaterials, graphene is the most suitable for fabrication of wet-spun fibers, owing to its liquid crystalline behavior (Cong et al., 2012; Xu Z. et al., 2013). In addition, graphene offers an array of peculiar properties (such as extraordinary electronic transport properties, thermal conductivity, and mechanical stiffness), which make them a unique candidate for development of conductive platforms (Stankovich et al., 2006; Chen et al., 2008). Graphene also showed to have the ability to interface/interact with the biomolecules, cells, and tissues, which further expanded the application of this nanomaterial in various biomedical fields (Zhang et al., 2012; Liu et al., 2013), ranging from tissue engineering to drug delivery. For instance, Ku et al. studied the effect of graphene oxide (GO) on mouse myoblast C2C12 cells, and their results revealed that myogenic differentiation was markedly enhanced on GO, which resulted from serum protein adsorption and nanotopographical cues (Ku and Park, 2013). Nevertheless, the biocompatibility of graphene is still a subject of debate (Zhang et al., 2011; Kiew et al., 2016), and specifically, issues such as its hemocompatibility, inflammation responses, and clearance pathways are major obstacles in the way of transition of graphene from laboratory to clinic (Kurapati et al., 2016). In addition, wet-spun graphene fibers often suffer from low flexibility and weak mechanical properties, which further hinder their practical application in the engineering field (Dong et al., 2012; Xiang et al., 2013).

Consequently, in this study, with the aim of developing a bio-friendly, conductive, and robust platform, we have utilized graphene nanosheets alongside the highly biocompatible alginate to institute composite fibers *via* the well-established wet-spinning technique. The resulting composite biofibers showed to have great flexibility and mechanical properties. Most remarkably, these composite fibers possessed a high level of electrochemical properties and showed a good level of cellular biocompatibility when tested against myoblast cell lines. Given the favorable properties of these nanocomposite biofibers, they could be used as scaffolding materials for tissue engineering applications.

## Experimental

### Materials

Alginic acid sodium salt from brown algae (medium viscosity) and graphene nanosheets (with electrical conductivity of >10^3^ S/m) were purchased from Sigma-Aldrich. Calcium chloride (CaCl_2_) and ethanol were purchased from Chem-Supply.

### Preparation of Spinning Solution

To prepare alginate (Alg) fiber, a spinning solution containing 3% (w/v) alginic acid in distilled water was prepared. To produce hybrid alginate/graphene (Alg/G) naocomposite fibers, 8% (wt%) of graphene nanosheets was added into the alginate solution (3% wt) under constant stirring and sonication. The mixture was further stirred and sonicated for 24 h to ensure homogeneous dispersion of graphene nanosheets into the polymer matrix.

### Fiber Spinning

Single alginate and nanocomposite Alg/G biofibers were fabricated using a coagulation bath of H_2_O/ethanol (1:1) containing 3% CaCl_2_ (w/v). Both types of fibers were fabricated by simply extruding the corresponding spinning solutions (50 ml/h) into the coagulation bath using a blunt needle (gauge 19).

### Instrumentation

All rheology experiments were conducted on a Physica MCR 301 Rheometer (Anton Paar) in parallel plate geometry (50-mm disk, 0.097-mm measuring distance) and at room temperature (23°C). Flow experiment was performed to evaluate the viscosity of polymer solution (shear rate varying from 1 to 100 s^−1^). Fourier transform infrared (FTIR) spectra were measured between 700 and 4,000 cm^−1^ on a Shimadzu IRPrestige-21 infrared spectrometer with internal beam equipped with standard detector with mirror speed of 2.8. The spectra were obtained using attenuated total reflection (ATR) on the fibers with resolution of 8 and number of scans of 30, on a transmittance mode. Raman spectra were recorded on a Jobin Yvon Horiba HR800 Raman system using a 632-nm laser line and a 300-line grating. The weight loss of the biofibers was obtained by thermogravimetric analysis (TGA; Mettler Toledo-SDTA851) on 10 mg of samples with heating rate of 5°C/min under a nitrogen atmosphere, between temperatures of 30° and 600°C. The morphology of fibers, and surface and cross-sectional structure were examined using a JSM-6490LV scanning electron microscope (SEM) and Leica M205A microscope. For SEM imaging, the fibers were cut into small pieces and inserted into holes that had been pre-drilled into a small brass block. The block containing the mounted fibers was then immersed into liquid nitrogen for about 45 s and a liquid nitrogen cooled razor blade was run across the surface of the block to fracture the fibers. The block was then quickly transferred to the low-voltage SEM (LVSEM) for examination. SEM images were taken in high vacuum (HV) mode at 15-kV operating voltage and a spot size setting of 60. The mechanical properties of fibers were assessed using a dynamic mechanical tester (EZ-L tester from Shimadzu) at 10 mm/min *via* 50- and 10-N load cells for dry and wet fibers, respectively. The swelling properties of the hydrogel fibers were determined by examining their water uptake capacity. The hydrogel fibers were incubated in simulated body fluid (SBF) at 37°C and allowed to fully swell. The swelling ratio was calculated using the equation (Ws – Wd)/Wd, where Ws represents the weight of the swollen hydrogel fibers and Wd represents the weight of the dried hydrogel fibers at the beginning.

A three-electrode electrochemical cylindrical cell (15 × 50 mm) coupled to a Bioanalytical Systems (Model CV27) potentiostat was used for cyclic voltammetry. Dry Alg/G fibers at 20 mm were used as the working electrode with a Ag/AgCl reference electrode and a Pt mesh counter electrode. All cyclic voltammetric tests were performed in SBF. The electrical conductivity of the fibers was measured using an in-house built, four-point probe. The electrodes consisted of four parallel rods at a spacing of 0.33 cm; the fibers were connected to the parallel rods using silver paint (obtained from SPI). A constant current was applied between the two outer electrodes using a potentiostat/galvanostat (Princeton Applied Research Model 363). The potential difference between the inner electrodes was recorded using a digital multimeter 34401A (Agilent).

### Cytotoxicity and Cell Morphology Studies

Murine C2C12 myoblasts cells, purchased from the European Collection of Cell Cultures (ECACC; catalog no. 91031101), were cultured in Dulbecco's modified Eagle medium (DMEM; Sigma-Aldrich, St. Louis, MO, USA) supplemented with 10% (v/v) fetal bovine serum (FBS; Sigma-Aldrich, St. Louis, MO, USA) and penicillin–streptomycin (1% v/v) and maintained at 37°C in the presence of 5% CO_2_ and 95% air. When ~70% confluency was reached, the cells were detached by using 0.25% (w/v) trypsin/0.1% (w/v) EDTA, and they were either subcultured or used to set up the experiments. The cytotoxicity and cell viability of fibers were evaluated by a colorimetric Cell Counting Kit-8 (CCK-8; Dojindo Laboratories Inc., Kumamoto, Japan). Briefly, cells (passage numbers between 4 and 6) were seeded in 96-well plates at a density of 5 × 10^3^ cells per well, to which 1 cm of fibers (either Alg or Alg/G) was added and subsequently allowed to culture for 1, 2, and 4 days. At each time point, fibers were taken out, and cells were washed twice with phosphate-buffered saline (PBS) and then incubated with Dulbecco's PBS (DPBS) solution, and then 100 μl of medium containing 10% CCK-8 solution was added to the cells and kept for a further 3 h. Then, the absorbance was read at the wavelength of 450 nm according to the manufacturer's instructions by a microplate spectrophotometer (Benchmark Plus, Tacoma, Washington, USA).

Fluorescent staining was employed to observe the cell morphology *via* confocal laser scanning microscopy (CLSM) (Zeiss LSM710, Carl Zeiss, Inc., Jena, Germany). Briefly, after day 4, the fibers were removed from the wells, and the cells were washed three times with DPBS. Then, the cells were incubated for 45 mi incubation at 37°C with a solution of rhodamine phalloidin [1:40 dilution in 0.1% (w/v) bovine serum albumin (BSA)]. The cells were washed three times in DPBS, and then the nuclei of the cells were stained with Hoechst (Thermo Fisher Scientific; 33342) and incubated for 15 min at 37°C. Then, cells were washed three times with DPBS before imaging. Furthermore, live staining, based on our previously published work, was implemented to evaluate the adhesion of cells to the surface of fibers (Mehrali et al., 2019). Briefly, the fibers were fixed in a 24-well plate using sterilized stainless steel rings. Next, cells (passage numbers between 4 and 6) were seeded on top of the fixed fibers in the 24-well plates at a density of 10 × 10^3^ cells per well and allowed to culture for 2 days. Next, the cells (on the fibers as well as cells growing on the tissue culture plastic underneath) were washed two times with DPBS and then stained with a calcein-AM (live cells) for 15 min at 37°C. The samples were washed twice with DPBS before the image is captured. Of note, to encourage the adhesion of cells to the fiber surface, we have coated the samples with a thin layer of collagen.

### Statistical Analysis

Statistical significance of treatment groups as compared with control groups was determined using a two-way ANOVA with a Bonferroni post-test or unpaired students multiple *t*-test (GraphPad Prism V 6.0; San Diego, CA, USA). *P* values < 0.05 were considered statistically significant. Values are reported as the average ± standard deviation.

## Results and Discussion

### Viscometry of Spinning Solution

**Figure 1:**
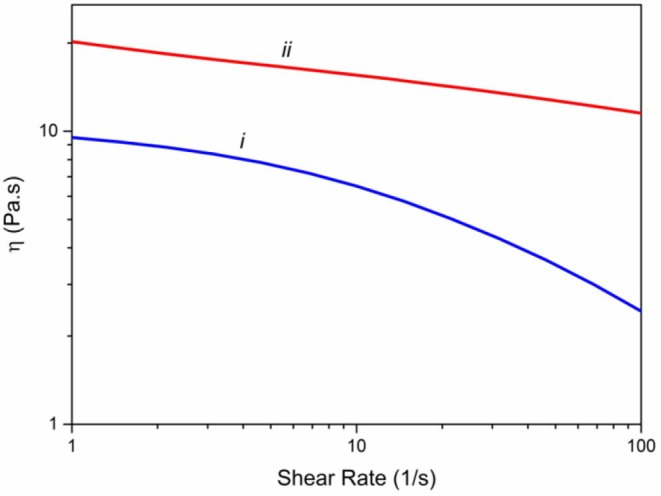
Viscometry of spinning solutions including (i) alginate 3% (w/v) and (ii) hybrid alginate–graphene (3 and 8% w/v, respectively).

Rheological properties of the spinning solutions are essential factors in determining the mechanical properties of the yielding fibers (Mirabedini et al., 2016a). Consequently, we have measured the viscosity of alginate solution (3%) and Alg/G solution (8% wt) in a ranges of 1–100 s^−1^ of shear rate (Figure 1). Accordingly, both solutions showed a shear thinning behavior; however, graphene-containing alginate solution experienced a much lower drop in its viscosity, a phenomenon that was observed previously with Alg/G composites (Li et al., 2016). In addition, graphene-containing solution showed a higher level of viscosity over the entire shear rate range, which was reported to be a consequence of attachment of alginate chains onto the surface of graphene sheets (Liu and Li, 2017).

### Morphology of As-Spun Fibers

**Figure 2:**
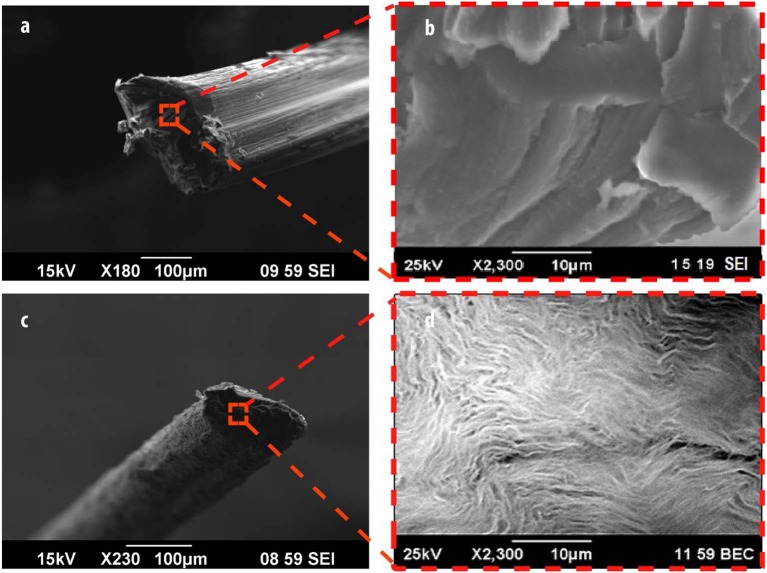
Morphological analysis of fibers, including scanning electron microscope (SEM) images of alginate **(a,b)** and alginate/graphene **(c,d)** fibers.

SEM imaging was utilized to assess the surface morphology of alginate (Figures 2a,b) and Alg/G fibers (Figures 2c,d). As-spun fibers were cut in the middle, and their cross section was further analyzed. Accordingly, Alg fibers showed a smooth surface corresponding to soft polymeric nature of alginate, whereas Alg/G fibers showed a layered dense structure with rough surface as a result of graphene sheets (Li et al., 2014). In addition, Alg/G fibers possessed an average diameter of 186 μm, whereas pure Alg fibers had an average diameter of 243 μm. This difference in fiber diameter is a consequence of intermolecular interactions between graphene and alginate (in correspondence with viscometry data), which leads to formation of a more compact structure. The SEM images of Alg/G fibers also revealed the homogenous dispersion of graphene sheets throughout the alginate polymeric network.

### Physiochemical Characterization of Fibers

**Figure 3:**
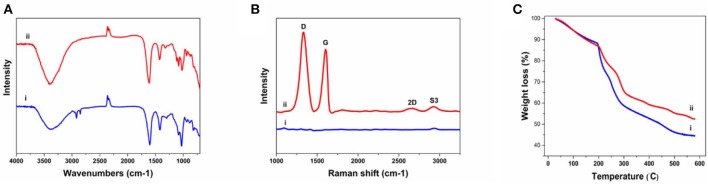
Physiochemical characterization of as-prepared fibers including **(A)** Fourier transform infrared (FTIR) spectra of (i) alginate and (ii) alginate/graphene, **(B)** Raman spectra of (i) alginate and (ii) alginate/graphene fibers, **(C)** thermogravimetric analysis (TGA) of (i) alginate and (ii) alginate/graphene fibers.

The FTIR was implanted to further characterize the chemical composition of the fibers (Figure 3A). The Alg fibers showed the characteristic peaks of alginic acid at 3,380, 1,600, 1,418, and 1,028 cm^−1^, corresponding to OH stretching vibration, symmetric, and asymmetric stretching vibration of carboxylate salt group, and stretching vibration of C–O–C groups, respectively (Zheng et al., 2016). The Alg/G fibers showed a similar absorption pattern to alginate, but the appearance of peaks between 1,050 and 1,250 cm^−1^ (C–O–C stretching vibration) and an increase in intensity of peak at 3,380 cm^−1^ (–OH stretching vibration) were assigned to graphene functional groups (Li et al., 2018). To further investigate the state of graphene sheets in the Alg/G nanocomposite fibers, we have conducted Raman spectroscopy (Figure 3B). Accordingly, the Raman spectrum of Alg fibers did not show any peak, whereas the Alg/G nanocomposite fibers showed the characteristic peaks of graphene at 1,332, 1,600, 2,655 (2D band), and 2,926 cm^−1^ (S3 band), corresponding to D vibration band arising from the breathing mode of j-point phonons of A_1g_ symmetry, G vibration band arising from the E_2g_ phonon of the sp^2^ C atoms, 2D band as an indicator of the number of graphene layers, and S3 band derived from the D–G peak combination, respectively (Johra et al., 2014; Mehrali et al., 2016). In addition, the D band to G band intensity ratio (*I*_D_/*I*_G_) was measured to be 1.21, suggesting that graphene was partially reduced in the fabrication process, which led to a decrease in the average size of the sp^2^ domains (Stankovich et al., 2007).

We also compared the thermal properties of alginate and Alg/G fibers using TGA (Figure 3C). The results showed that both fibers undergo three-stage thermal degradation processes: (i) the loss of volatile products through dehydration (from room temperature to 200°C), (ii) the thermal degradation of the polymer (200–300°C), and (iii) the carbonization process (above 300°C). As-prepared Alg/G fibers indicated that the degradation and proceeding carbonization processes happened at slightly higher temperatures, when compared with that of Alg fibers, which showed that graphene nanosheets enhanced the thermal stability of alginate and retarded the pyrolysis of the composite fibers. This might be associated with the graphene layers restraining the movement of the alginate polymeric chains, impeding the thermal decomposition process, and increasing the needed energy of thermal decomposition (Li et al., 2018). Lastly, the mass fraction of the graphene in the composite fibers was evaluated by comparing the TGA graph of alginate and alginate–graphene at 580°C, which showed that about 8 wt% of the fibers was composed of graphene nanosheets.

**Figure 4:**
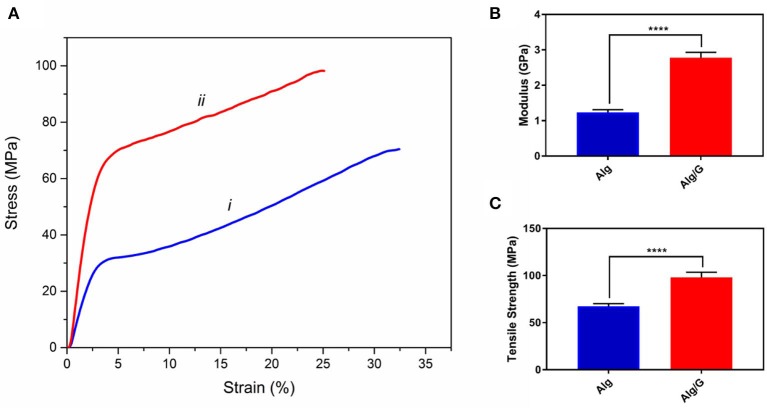
Mechanical properties of as-prepared (i) alginate (Alg) and (ii) alginate-graphene (Alg/G) nanocomposite fibers including **(A)** stress-strain curve, **(B)** modulus, and **(C)** tensile strength (*n* = 4, mean ± SD, ^****^P < 0.0001).

The mechanical properties of soft conductors are one of the most essential traits of these systems, especially when it comes to practical applications such as tissue engineering or biosensors (Mehrali et al., 2018). Consequently, we have measured the mechanical properties of the as-prepared fibers, and the results are shown in Figure 4. As can be seen from Figure 4, the addition of graphene to alginate led to an increase in tensile strength (from 68 to 98 MPa) and modulus (from 1.26 to 2.77 GPa) of the resulting fibers. This could be a result of graphene–alginate interactions (functional groups on the edges of graphene sheets and the hydroxyl groups on the alginate backbone) that enable the load transfer from the matrix to single-layer graphene sheets enhancing tensile strength and also helping to absorb more energy before fracture (Li et al., 2014; Hu et al., 2016). The tensile strength and modulus values obtained in this study were slightly lower than the reported value in the literature for similar fibers (tensile strength and modulus normally ranging from 50 to 600 MPa and 1–40 GPa, respectively), which could be result from low concentration of alginate as well as graphene nanosheets in our fibers (Xu and Gao, 2014; Ma et al., 2015). Most remarkably, Alg/G fibers in this study exhibited an elongation at break of 25%, which put these fibers among the most flexible graphene-containing fibers in the literature. Wet-spun graphene-containing fibers normally possess an ultimate tensile strain of <10%, which further distinguishes our work from similar studies (Cong et al., 2012; Chen et al., 2013; Xu and Gao, 2014; Hu et al., 2016; Fu et al., 2019). Also, Alg/G fibers exhibited a lower strain at break than did Alg fibers, which was due to contribution of graphene in the load bearing.

**Figure 5:**
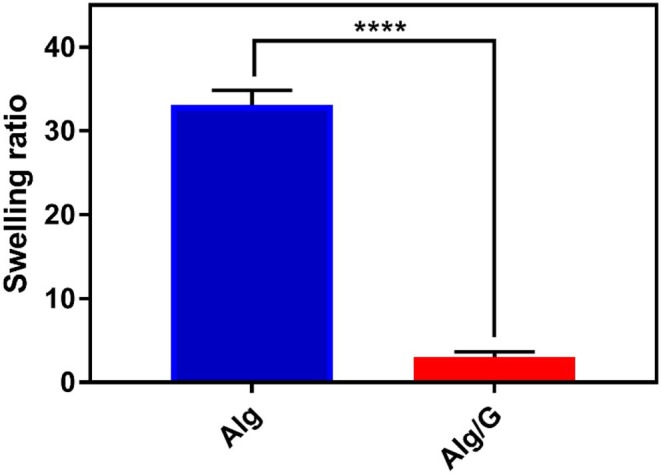
Swelling ratio of as-prepared fibers in simulated body fluid (SBF) including alginate (Alg) and alginate–graphene (Alg/G) nanocomposite fibers (*n* = 4, mean ± SD, ^****^P < 0.0001).

The practical application of hydrogels is often hindered by the difficulty in controlling their temporal change in shape after the installation (Kamata et al., 2015; Talebian et al., 2018). Consequently, controlling the swelling of hydrogels appeared as an important step toward their clinical realization. In line with this, we have measured the swelling ratio of the as-spun fibers in SBF, and the results are shown in Figure 5.

Accordingly, Alg/G composite fibers possessed a much less swelling ratio (2.9) than did single Alg fibers (33.07). This is probably due to graphene–alginate interactions that also led to an increase in mechanical properties of this composite fiber (Liu S. et al., 2017; Peng et al., 2017). The obtained swelling ratio value for Alg/G fibers in our study is by far among the lowest values reported in the literature (Peng et al., 2017, 2018; Zhao et al., 2017), which made these fibers not easily destroyable by the swelling force.

**Figure 6:**
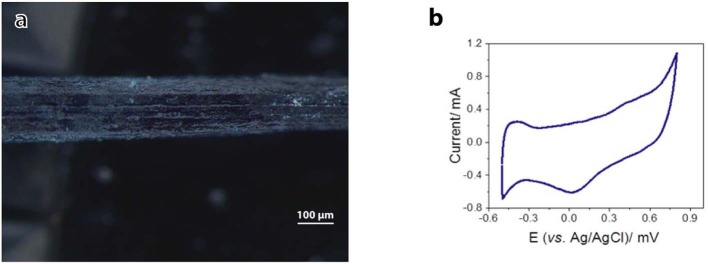
Electrical conductivity of alginate–graphene fibers. **(a)** Light microscope image of the fiber used as the working electrode. **(b)** Cyclic voltammograms of alginate–graphene fibers. Potential was scanned between −0.5 and +0.8 V (vs. Ag/AgCl) in simulated body fluid (SBF) at 100 mV/s.

Electrical conductivity and electrochemical properties of the as-prepared Alg/G nanocomposite fibers have been investigated to evaluate their capabilities as a smart biofiber for electrical stimulation. Cyclic voltammetry for the as-prepared Alg/G fibers in SBF was carried out to evaluate their electrochemical performance. It can be seen from Figure 6 that the CV curve of the Alg/G fibers displayed a box-like shape superimposed with a pair of Faradaic peaks in the potential range of −0.45 to +0.15 mV, which is caused by the reversible redox reaction of oxygen-containing groups on the graphene sheets, and it is an indication that redox reactions are occurring owing to the conducting fibers providing a broad range of energy states (Xu Y. et al., 2013; Ates et al., 2018). In addition, the electrical conductivity of the as-spun Alg/G nanocomposite fiber was 2 S/m. The obtained value for electrical conductivity of our fibers is not comparable with the values reported in other studies (Jalili et al., 2013; Xu et al., 2016), mostly owing to low concentration of graphene in our composite fibers, which was intentionally chosen to yield flexible fibers with an acceptable level of conductivity.

### *In vitro* Studies

**Figure 7:**
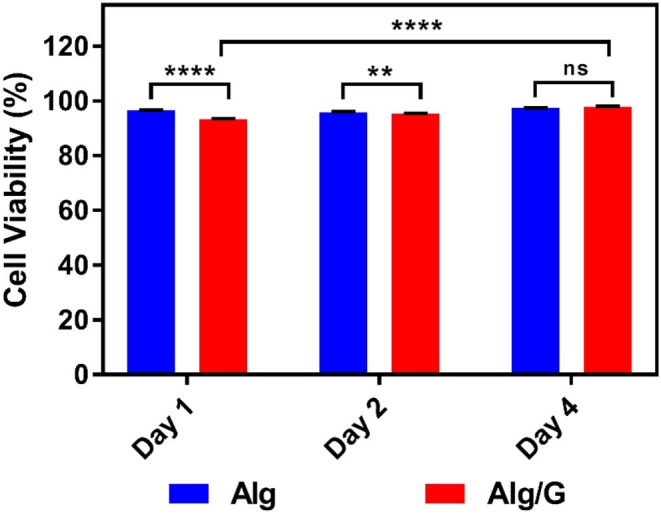
C2C12 cell viability studies treated with as-prepared alginate (Alg) and alginate–graphene (Alg/G) fibers after different time points (1, 2, and 4 days) (*n* = 4, mean ± SD, ns *P* = 0.1196, ^**^P = 0.0036, ^****^P < 0.001).

**Figure 8:**
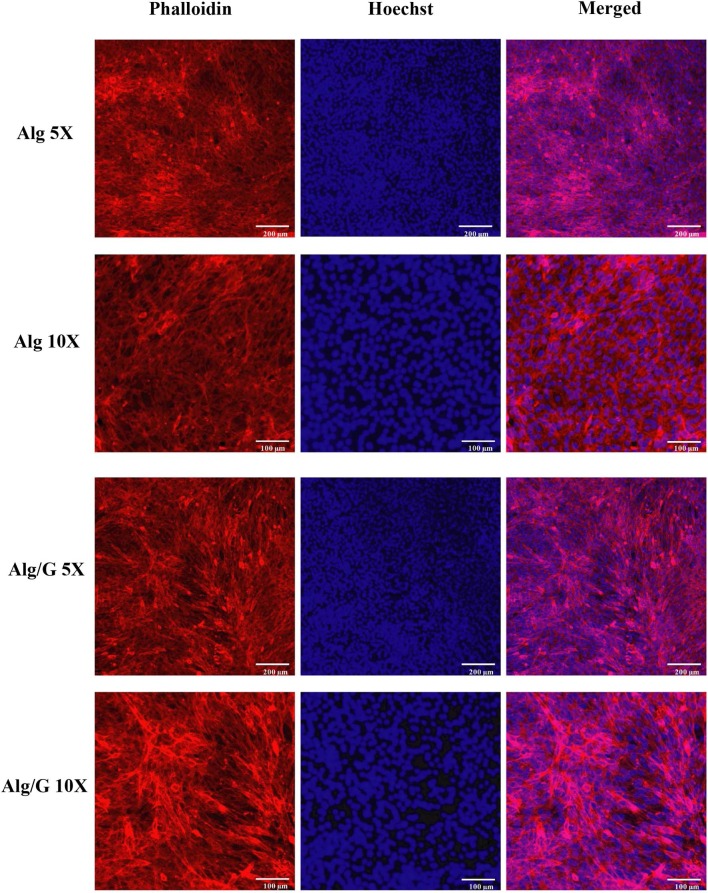
C2C12 spreading and viability after being treated with the as-prepared alginate (Alg) and alginate–graphene (Alg/G) nanocomposite fibers. Phalloidin (red)–Hoechst nuclear staining (blue) of the cells after 4 days of culture with different magnifications.

In order to investigate the biocompatibility of hybrid Alg/G fibers, murine myoblast cell line (C2C12) was used and its cell viability and cell morphology were compared with those of single Alg fibers (Figures 7, 8, respectively). We have investigated the biocompatibility of the fibers by using a colorimetric CCK-8, and the results are depicted in Figure 7. The cell viability results showed that Alg/G fibers maintained a good level of cell viability even after 4 days of culture. At day 1, cells treated with Alg/G (compared with Alg fibers) showed a lower level of viability, which could be a consequence of released graphene nanosheets that can be taken up by the cells, causing low toxicity (Patel et al., 2016). Interestingly, the viability of cells treated with Alg/G fibers started to increase as the time passes, to a point where after 4 days of culture, no significant difference was observed between these cells and the ones treated with Alg fibers. This further proved that Alg/G fibers were not toxic to myoblasts but rather increased their proliferation, a phenomenon reported previously in the literature (Ku and Park, 2013). Furthermore, the Hoechst staining revealed that both Alg and Alg/G fibers had a good level of biocompatibility against the cells, as indicated by the number of viable cell nucleus stained by Hoechst (Figure 8). In addition, they did not induce any unwanted morphological features in the C2C12 cells and kept the characteristic spindle-shaped morphology in the growth media, as indicated by phalloidin staining of actin filaments. Furthermore, C2C12 cells treated with Alg/G fibers were fused to a multinucleate elongated shape, an early indication of myogenic differentiation of myoblasts caused by the presence of graphene in these fibers (Kim M. J. et al., 2015; Lee et al., 2016).

**Figure 9:**
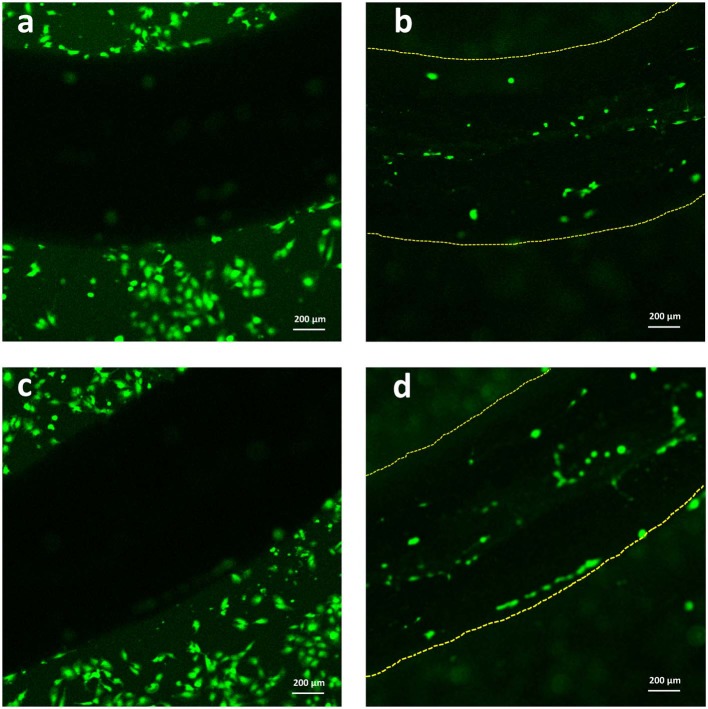
Live staining of C2C12 cells after 2 days of culture **(a,c)** around the Alg/G fibers and **(b,d)** on the surface of Alg/G fibers.

Lastly, with the aim of evaluating the adhesion of C2C12 cells onto the Alg/G fibers, we have utilized a live cell staining method (Figure 9). The results showed that cells started to migrate onto the surface of fibers only after 48 h of culture. The lower cell density on the fibers (compared with the area adjacent to the fiber) could be due to lack of bioactive functional groups (amines or sulfates) in alginate, which hindered further migration of cells onto the fibers (Dinoro et al., 2019). This shows that these fibers might have the potential to be also used as conductive scaffolds, if modified with proper functional groups, to encourage the regeneration of cells.

## Conclusions

In this study, with the aim of establishing a flexible, robust, biocompatible, and electrical conducting hydrogel, we have used a simple wet-spinning method to fabricate nanocomposite fibers from a mixture of alginate and graphene nanosheets. The resulting nanocomposite biofibers showed to have better mechanical properties, lower swelling ratio, and higher thermal stability than did single fibers made from pure alginate. Most remarkably, these composite biofibers possessed excellent electrochemical properties, and when tested against C2C12 myoblast cell lines, they showed high level of biocompatibility. Overall, these fibers hold immense promise for use as soft conductors in tissue engineering, and future work will include the application of these fibers as smart biopolymer scaffolds in multi-component systems to allow electrical stimulation of cells toward their maturation.

## Data Availability Statement

All datasets generated for this study are included in the article/supplementary materials.

## Author Contributions

All authors listed have made a substantial, direct and intellectual contribution to the work, and approved it for publication.

### Conflict of Interest

The authors declare that the research was conducted in the absence of any commercial or financial relationships that could be construed as a potential conflict of interest.
